# Effect of ropivacaine with and without intrathecal fentanyl for spinal anesthesia among patients with scorpion bite history: A case control study

**DOI:** 10.6026/973206300211577

**Published:** 2025-06-30

**Authors:** Sonali Tripathi, Vineet Mandrah, Ritesh Upadhyay, Ashwini Kumar Patel

**Affiliations:** 1 Department of Anaesthesiology, Chhindwara Institute of Medical Sciences, Chhindwara, Madhya Pradesh, India; 2 Department of General Surgery, Chhindwara Institute of Medical Sciences, Chhindwara, Madhya Pradesh, India; 3 Department of Community Medicine, Chhindwara Institute of Medical Sciences, Chhindwara, Madhya Pradesh, India

**Keywords:** Intrathecal fentanyl, ropivacaine, spinal anesthesia, scorpion bite, pain management, analgesic efficacy

## Abstract

Effect of ropivacaine with and without intrathecal fentanyl for spinal anesthesia among patients with scorpion bite is of interest. Patients in the fentanyl-ropivacaine combination group had faster onset of sensory blockade (5.2±1.1 versus 7.8±1.3 minutes, p<0.001) and significantly longer analgesia duration (290±15 versus 180±12 minutes, p<0.001) than patients in the ropivacaine-alone group. Hemodynamic stability and the incidence of adverse effects were similar between the two groups. These results indicate that intrathecal fentanyl enhances the efficacy of ropivacaine in this distinct patient population without compromising safety.

## Background:

In the tropical and subtropical regions, scorpion envenoming is an important public health problem through its systemic manifestations by neurotoxins from the venom [1]. Such neurotoxins could be ion channel interfering or neurotransmitter releasing that can alter the perception of pain or reaction to anaesthetics [2]. There is a case report of a patient who has been stung by scorpions before with some specific features about anesthesia, which makes the challenge in the management of spinal anesthesia for this population of patients [3]. Ropivacaine is one of the most widely used long-acting amide local anesthetics for spinal anesthesia due to favorable sensory-motor differentiation and lower cardiotoxicity than bupivacaine [4]. Onset time and duration may not always meet the clinical needs, especially in cases with altered neurophysiology [5]. Intrathecal fentanyl, being a lipophilic opioid agonist, has been used as an adjunct to improve the quality of spinal anesthesia through rapid onset and prolonged analgesia [6]. It was previously shown that adding fentanyl to local anesthetics has beneficial effects in general populations [7]. However, the literature on the efficacy of the available information in patients with a history of having envenomation by a scorpion is sparse. Understanding the interaction between the neurotoxic effects of scorpion venom and anesthetic agents is important for optimizing perioperative care in this unique patient group [8]. Therefore, it is of interest to evaluate the effectiveness of intrathecal fentanyl added to ropivacaine as compared to ropivacaine alone for spinal anesthesia in a patient population with a history of previous scorpion bite. This would improve the onset and duration of anesthesia without increasing adverse effects; that is my hypothesis.

## Materials and Methods:

This was a case-control study conducted at C.I.M.S. Chhindwara, MP, between January and June 2024, after obtaining institutional review board ethical approval (Approval No. CIMS/EC/2024/8504). Informed written consent was taken from all patients. Sixty patients aged between 18 and 60 years were enrolled who had a history of scorpion envenomation at least one year back and were posted for elective lower limb surgery under spinal anesthesia. Volunteers were placed ASA physical status I or II. Patients with allergy to the drugs under investigation, coagulopathy, local infection at the injection site and neurological deficits were excluded from this study. Randomization of 30 patients into two groups was done in a computer-generated table. The three-millilitre 0.75% isobaric ropivacaine was administered intrathecally to Group R and to Group RF, 3 mL of 0.75% isobaric ropivacaine was added with 25 µg of fentanyl. Spinal anesthesia was instituted at the L3-L4 interspace in asepsis using a 25G Quincke needle and standard monitors were applied, with the baseline hemodynamic parameters being noted. These were collected regarding several parameters, including sensory blockade time and the onset and duration of sensory blockade, measured at T10 and regression to S1 dermatome, respectively; likewise, the onset and the duration of motor blockade using the Bromage scale, hemodynamic parameters like heart rate and blood pressure, adverse effects such as nausea, vomiting, pruritus and respiratory depression. It used SPSS version 25 for analysis; it applied an unpaired t-test on quantitative variables and a chi-square test for qualitative variables and a p-value of less than 0.05 was considered to be statistically significant.

## Results:

The tables in the study give important insights into the findings. Table 1 (see PDF) shows that the two groups were demographically similar, with no significant differences in age, weight, height, or ASA physical status. Table 2 (see PDF) indicates a quicker onset and longer duration of motor blockade in the fentanyl-ropivacaine group than in the ropivacaine-alone group, which shows the improved efficacy of the combination. Table 3 (see PDF) shows incidence of adverse effects. There is a low incidence of adverse effects, with mild pruritus being noted in a few patients in the fentanyl-ropivacaine group, but no significant differences in safety outcomes between the groups. These tables collectively underscore the advantages of adding fentanyl to ropivacaine for spinal anesthesia in this patient population. The figures in the study provide key visual insights into the findings. Figure 1 (see PDF) depicts the onset of sensory blockade, demonstrating a significantly faster onset in the fentanyl-ropivacaine group compared to the ropivacaine-alone group. Figure 2 (see PDF) illustrates hemodynamic parameters over time, showing no significant differences in heart rate and blood pressure between the two groups, thereby confirming the hemodynamic stability of both regimens. These figures together show that adding fentanyl to ropivacaine for spinal anesthesia in patients with a history of scorpion envenomation improves its efficacy and safety. Table 1 (see PDF) shows that both groups were demographically similar with no significant differences in age, weight, height, or ASA physical status, confirming baseline comparability. Table 2 (see PDF) highlights that the addition of fentanyl to ropivacaine significantly reduced the onset time and prolonged the duration of motor blockade compared to ropivacaine alone, indicating enhanced anesthetic efficacy. Table 3 (see PDF) presents the incidence of adverse effects, which were minimal and comparable between groups, with only mild pruritus observed in a few patients receiving fentanyl, and no serious complications such as respiratory depression reported.

## Discussion:

This study demonstrates that the intrathecal addition of fentanyl to ropivacaine significantly increases the onset and prolongs the duration of both sensory and motor blockade in patients with a history of scorpion envenomation. The results agree with other studies in general populations, thus further strengthening the evidence that fentanyl is an effective adjuvant to local anesthetics in spinal anesthesia [6, 9]. In short, fentanyl increases the potencies of ropivacaine through synergism hence enhancing clinical outcome in cases that the patient has neurophysiologic functions probably modified due to the toxins caused by the venom. In detail, potent toxins are related to the venoms which act significantly on ionic channels of sodium potassium and calcium. Consequently, such toxins prevent further neurotransmission [10]. The venom may upregulate or mutate sodium channels, as shown in animal models, which may be the reason patients with a history of scorpion sting show slower or less complete blockade with local anesthetics alone [11]. In this study, the faster onset and prolonged duration of sensory and motor blockade by fentanyl are critical because they may counteract these neurophysiological changes. Ropivacaine is alone said to delay onset in some patient populations and this could possibly be enhanced in those who are exposed to neurotoxins such as scorpion venom. Fentanyl, being a lipophilic opioid, can easily cross the blood-brain barrier and penetrate neural tissue [12]. Its primary mechanism of action is on opioid receptors in the spinal cords substantial gelatinosa where it inhibits the transmission of pain. By synergistically interacting with ropivacaine, fentanyl may potentiate the local anesthetic effect by regulating the ion channels affected by the venom exposure. Therefore, the combined effect of both drugs is to achieve a better and longer blockade of the neuronal excitability affected by the venom and thus ensures better pain relief. Hemodynamic stability is an important consideration in spinal anesthesia, especially in patients who have been previously envenomed, as they are likely to have cardiovascular sequelae [8]. Scorpion stings can cause an exaggerated sympathetic response, leading to transient hypertension, tachycardia, or bradycardia. However, our study found no significant differences in heart rate or blood pressure between the two groups throughout the perioperative period, which is consistent with prior research [13]. This stability is particularly important to ensure patient safety since hemodynamic fluctuations can make anesthesia management complicated. Our study also puts further light on this minimalist cardiovascular impact of this additive effect found in safety profiles when ropivacaine is given in combination with fentanyl in this population. In both groups, side effects were infrequent and mild. Only two patients in Group RF felt pruritus. The extremely low incidence is consistent with the established safety profile of intrathecal fentanyl [14]. The lipophilic nature of fentanyl allows for rapid clearance from the cerebrospinal fluid, thus minimizing the risk of respiratory depression or other opioid-related side effects [12, 14]. This rationale further supports this combination in clinical practice, particularly in neurotoxic venom-exposed patients with altered pain and anesthetic responses. However, the study has limitations. The relatively small sample size and single-center design limit the generalizability of our findings. Larger, multicentric studies will now be needed in order to solidify the observations made with the use of intrathecal fentanyl in an individual with previous scorpion stings [13]. Whether recurrence of pathologic response or chronic pain emergence occurred was not delineated in regard to long term follow-up study. Further investigations should be emphasized on these outcomes so that a comprehensive comprehension of anesthesia handling in such an exclusive population would be achieved [15]. Overall, fentanyl added to intrathecal ropivacaine in history-taking patients increases both the rate of onset and duration of blockades of sensations as well as of motor ones with no harmful impact on patients' hemodynamic stability without increase in morbidity. Overall, this method has a promising effect in anesthesia performed via spinal technique on patients potentially whose neurophysiologic effects would be modulated by toxin activity of certain species of the venomous stings.

## Conclusion:

Addition of intrathecal fentanyl to ropivacaine improves anesthetic efficacy without reducing safety in a patient with scorpion envenomation.
